# Detection of the transforming *AKT1 *mutation E17K in non-small cell lung cancer by high resolution melting

**DOI:** 10.1186/1756-0500-1-14

**Published:** 2008-05-16

**Authors:** Hongdo Do, Benjamin Solomon, Paul L Mitchell, Stephen B Fox, Alexander Dobrovic

**Affiliations:** 1 Department of Pathology, Peter MacCallum Cancer Centre, Locked Bag 1, A'Beckett St, Melbourne, Victoria, 8006, Australia; 2 Department of Pathology, University of Melbourne, Parkville, Victoria, 3010, Australia; 3 Division of Haematology and Medical Oncology, Peter MacCallum Cancer Centre, Locked Bag 1, A'Beckett St, Melbourne, Victoria, 8006, Australia; 4 Ludwig Medical Oncology Department, Austin Hospital, Heidelberg, Victoria, 3084, Australia

## Abstract

**Background:**

A recurrent somatic mutation, E17K, in the pleckstrin homology domain of the *AKT1 *gene, has been recently described in breast, colorectal, and ovarian cancers. AKT1 is a pivotal mediator of signalling pathways involved in cell survival, proliferation and growth. The E17K mutation stimulates downstream signalling and exhibits transforming activity *in vitro *and *in vivo*.

**Findings:**

We developed a sensitive high resolution melting (HRM) assay to detect the E17K mutation from formalin-fixed paraffin-embedded tumours. We screened 219 non-small cell lung cancer biopsies for the mutation using HRM analysis. Four samples were identified as HRM positive. Subsequent sequencing of those samples confirmed the E17K mutation in one of the cases. A rare single nucleotide polymorphism was detected in each of the remaining three samples. The E17K was found in one of the 14 squamous cell carcinomas. No mutations were found in 141 adenocarcinomas and 39 large cell carcinomas.

**Conclusion:**

The *AKT1 *E17K mutation is very rare in lung cancer and might be associated with tumorigenesis in squamous cell carcinoma. HRM represents a rapid cost-effective and robust screening of low frequency mutations such as *AKT1 *mutations in clinical samples.

## Findings

### Genetic alterations in *AKT*

The human homologues of the viral oncogene *v*-*akt*, encode serine/threonine protein kinases which have a pivotal activity in the PI3K-related signalling pathway, affecting cell survival, proliferation and invasion [1]. The three highly homologous isoforms, *AKT1*, *AKT2 *and *AKT3 *are composed of three functional domains: an amino terminal pleckstrin homology (PH) domain, a central catalytic domain, and a carboxyl terminal regulatory domain with the hydrophobic motif. Deregulated AKT activity is a well-established genetic defect implicated in tumorigenesis [2]. On activation, AKT exerts its anti-apoptotic and proliferative cellular functions through its kinase activity on various substrates [3].

Frequent genetic alterations of the *AKT *genes, especially *AKT2*, have been demonstrated in human malignancies [4,5]. *AKT1 *amplification was initially identified in gastric cell lines [6] and has shown to be associated with cisplatin resistance in lung cancer cells through the mTOR pathway [7]. No *AKT1 *somatic mutation was described until Carpten *et al*. identified an E17K (c.49G>A) mutation in the pleckstrin homology domain from breast, ovarian and colorectal cancers [8]. Transforming activity was demonstrated both *in vivo *and *in vitro*. The mutation causes constitutive activation by localisation to the plasma membrane in a PI3K-independent manner.

It is of interest which tumour types other than breast, ovarian and colorectal cancers also show the recurrent E17K mutation. The mutation was not found in acute myeloid leukaemia and brain tumours [9,10]. In this study, we used high resolution melting (HRM) analysis to screen the *AKT1 *E17K mutation in a panel of 219 non-small cell lung cancer (NSCLC) tumour biopsies to determine if this mutation may occur in NSCLC.

## Methods

A total of 219 NSCLC samples were included in this study. Two hundred of these samples had previously been tested for *EGFR *and *KRAS *mutations. Our panel of samples have also been described in another recent study [20]. Of those, 141 were adenocarcinomas, 39 were large cell carcinomas, 14 were squamous cell carcinomas, and 25 were of unknown or other histology. Genomic DNA was extracted using the DNeasy Tissue kit (Qiagen, Hilden, Germany) following proteinase K digestion at 56°C for 3 days. This study was approved by the Ethics of Human Research Committee at the Peter MacCallum Cancer Centre (project number 03/90).

HRM primers were designed to screen the E17K mutation, generating a 78 bp product. The sequences of the primers were 5'-CGAGGGTCTGACGGGTAGAGTG-3' (forward) and 5'-GGCCGCCAGGTCTTGATGT-3' (reverse). PCR for HRM analysis was performed on the Rotor-Gene 6000 (Corbett Research, Sydney, Australia) in the presence of the fluorescent DNA intercalating dye, SYTO 9 (Invitrogen, Carlsbad, CA). The reaction mixture in a final volume of 20 μl was made using HotStarTaq (Qiagen) as follows; 1 × PCR buffer, 2.5 mM MgCl2, 200 nM of each primer, 5 ng of genomic DNA, 200 μM of dNTPs, 5 μM of SYTO 9, and 0.5 U of Taq polymerase. The cycling and melting conditions were as follows; one cycle of 95°C for 15 minutes; 50 cycles of 95°C for 10 seconds, 65°C for 10 seconds with an initial 10 cycles of touchdown (1°C/cycle), 72°C for 20 seconds; one cycle of 97°C for 1 minute and a melt from 70°C to 95°C with the temperature rising 0.2°C per second. All samples were tested in duplicate.

Samples with potential mutations identified by HRM analysis were sequenced. Each sample was reamplified with a new reverse sequencing primer to cover the entire coding region of the exon 4. The sequence of the reverse primer was 5'-CCCCAAATCTGAATCCCGAGA-3'. PCR was performed as follows; initial denaturation at 95°C for 15 minutes; 50 cycles of 95°C for 10 seconds, 60°C for 10 seconds, 72°C for 20 seconds with an initial 10 cycles of touchdown (1°C/cycle starting from 65°C); one cycle of 72°C for 10 minutes. 6 μl of the PCR products were purified with ExoSapIT (GE Healthcare, Little Chalfont, England) followed by sequencing using Big Dye Terminator v3.1 (Applied Biosystems, Foster City, CA) according to the manufacturer's protocol. The sequencing products were ethanol precipitated before running on a 3100 Genetic Analyser (Applied Biosystems). The sequencing data was analysed using Sequencher 4.6 (Gene Codes Corporation, Ann Arbor, MI).

## Results

**Figure 1:**
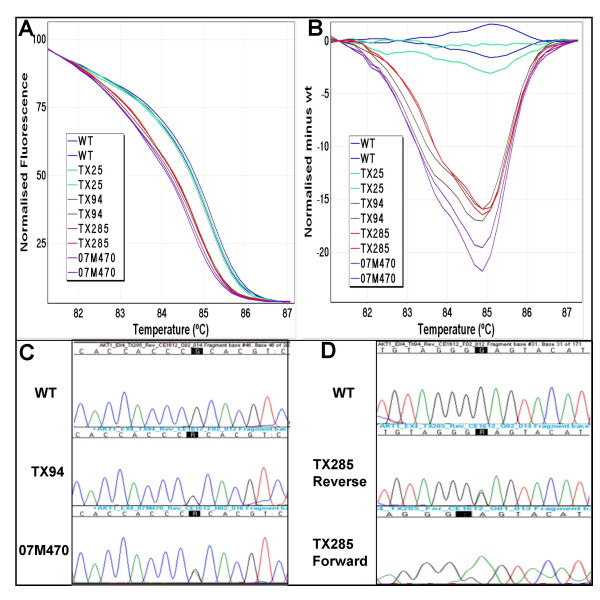
**High resolution melting analysis and sequence traces**. **Panel A**: **Normalised plot of *AKT1 *exon 4**. Melting profiles of the wild-type control (WT) and four samples (TX25, TX94, TX285, 07M470) are shown. The melting profile of TX25 is similar to that of the wild-type control. TX94, TX285 and 07M470 show different melting profiles than the wild-type control. **Panel B**: **Difference plot of *AKT1 *exon 4**. Melting profiles of each sample were normalised against the wild-type control (WT). TX94, TX285 and 07M470 show distinctively different melting curves. **Panel C**: **Sequencing of the HRM positive samples**. These identified a rare SNP, IVS3-12G>A, from both TX94 and 07M470. **Panel D**: **Sequencing identifies the E17K (c.49G>A) mutation**. TX285 was positive for the E17K (c.49G>A) mutation. Sequencing was carried out in both directions to confirm the presence of the mutation.

HRM analysis on a panel of 219 NSCLC samples identified four HRM positive samples which displayed aberrant melting curves compared with wild-type. Their melting curves were clearly separated from that of wild-type, suggesting that sequence changes were present in those amplicons (Figure 1). Subsequent sequencing of those samples revealed an E17K mutation in one sample and a rare intronic SNP c.47-12G>A (rs17846816) in the remaining three samples. The E17K mutation was detected in one of the 14 squamous cell carcinomas, and was absent in the 141 adenocarcinomas and 39 large cell carcinomas as well as the 25 samples of unknown or other histology.

## Discussion

High resolution melting (HRM) is a methodology that can detect sequence changes in amplicons through monitoring of the fluorescence of a double stranded DNA binding dye which dissociates from the DNA as it denatures with increasing temperature. It is an in-tube method which can be performed in a fast, simple, and robust manner. If sequence changes, either SNPs and/or mutations, are present within the amplicon, they cause a difference in the melting profile compared with the wild-type.

A range of alternative methodologies are available for screening monomorphic mutations such as the c.49G>A (E17K). These include PCR-RFLP [11], allele specific competitive blocker polymerase chain reaction [12,13] and TaqMan "genotyping" [14,15]. Each methodology has its own particular advantages and disadvantages. The disadvantages of these methodologies are that they detect only specified mutations (competitive blocker, TaqMan) and/or require post-PCR handling of the product (PCR-RFLP, competitive blocker) or relatively expensive probes for visualisation (TaqMan). However, HRM is capable of detecting any sequence changes present within the amplicon tested, and thus can screen not only the specified mutation but also the whole amplified sequence. Furthermore, the use of a fluorescent intercalating dye allows HRM to be performed in-tube, which eliminates post-PCR handling.

**Figure 2:**
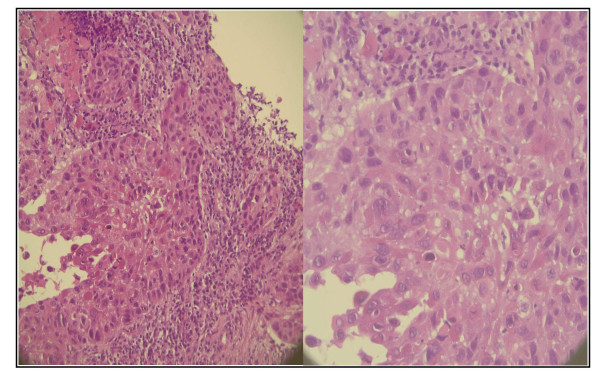
**Hematoxylin and eosin stained section of the tumour with an *AKT1 *E17K mutation**. A 5 μm tumour section was stained with hematoxylin and eosin. The tumour was histologically diagnosed as a squamous cell carcinoma. Two magnifications are shown.

In this study, the E17K (c.49G>A) mutation in exon 4 of the *AKT1 *gene was screened by HRM with primers generating a short amplicon of 78 bp suitable for the screening of formalin-fixed paraffin-embedded samples. By HRM screening and subsequent sequencing, we found the *AKT1 *E17K mutation in one of the 219 NSCLC samples. The tumour sample with the transforming mutation was collected from a female diagnosed with NSCLC at the age of seventy. The tumour biopsy (2 × 3 mm size) was taken from the left upper lobe and was histologically confirmed as squamous cell carcinoma (Figure 2). No mutation was found in *EGFR *exons 18 to 21 and *KRAS *exon 2 in this sample. More cases need to be studied to determine whether this mutation is recurrent in squamous cell carcinomas.

Association of AKT activation with resistance to chemotherapeutic agents has been studied in lung and breast cancers. *AKT1 *amplification was shown to be a cause of cisplatin resistance in lung cancer cells [6]. In another study, constitutive activation of AKT was found in over 90% of NSCLC cell lines (16/17) and its activation contributed to both PI3K inhibitor (LY294002) and radiation resistance [16]. In breast cancer cell line studies, AKT activation markedly increased resistance to microtubule-directed agents as well as trastuzumab and tamoxifen treatment [17,18]. Furthermore, the E17K was shown to alter the sensitivity to an allosteric kinase inhibitor but not to ATP competitive inhibitors. The finding of this recurrent *AKT1 *mutation in various tumours suggests that AKT may be an attractive therapeutic target for some patients.

In conclusion, this study shows that the transforming *AKT1 *E17K mutation occurs at a very low frequency in NSCLC. It may be restricted to squamous cell carcinoma where its frequency is likely to be higher. In addition, HRM is an effective screening method for this mutation due to its low incidence.

### Note added in proof

A second study has appeared since this MS was submitted for review. Malanga *et al*. examined a panel of 105 NSCLC by sequencing and observed E17K mutations in two of the 36 squamous cell carcinomas but in no other NSCLC subtype [19]. These results and our results considered together indicate that the E17K mutation is probably limited to squamous cell carcinomas in NSCLC.

## Authors' contributions

HD participated in development of the assays carried out the HRM studies, prepared the figures and wrote the manuscript. BS, SF and PM discussed the results and contributed to the manuscript. This study was initiated as part of a grant to PM and AD. AD initiated the project, was responsible for primer design, participated in the data analysis, and co-wrote the manuscript. All authors read and approved the final manuscript.
